# Antioxidant and Anti-Cytotoxicity Effect of Phenolic Extracts from *Psidium guajava* Linn. Leaves by Novel Assisted Extraction Techniques

**DOI:** 10.3390/foods12122336

**Published:** 2023-06-10

**Authors:** Md. Anisur Rahman Mazumder, Arif Tolaema, Pongpasin Chaikhemarat, Saroat Rawdkuen

**Affiliations:** 1 Food Science and Technology Program, School of Agro-Industry, Mae Fah Luang University, Chiang Rai 57100, Thailand; anis_engg@bau.edu.bd (M.A.R.M.); 5931401067@lamduan.mfu.ac.th (A.T.); 5931401039@lamduan.mfu.ac.th (P.C.); 2 Department of Food Technology and Rural Industries, Bangladesh Agricultural University, Mymensingh 2202, Bangladesh; 3 Unit of Innovative Food Packaging and Biomaterials, School of Agro-Industry, Mae Fah Luang University, Chiang Rai 57100, Thailand

**Keywords:** anti-cytotoxicity, guava leaf, extraction, ethanol/water, antioxidant, anticancer, morin

## Abstract

Phytochemicals (PCs) are gaining popularity due to their antioxidant effects and potential protection against infection, cardiovascular disease, and cellular metabolic activity. These PCs must be retained as much as possible during extraction. This research focused on the extraction of PC from *Psidium guajava* Linn. leaves due to higher antioxidant potential. Solvent extraction (SE), microwave-assisted extraction (MAE), and ultrasound-assisted extraction (UAE) using distilled water (DW) or 60% (*v/v*) ethanol/water (ET) were used for the extraction of PC. ET shows higher total phenolic (TPC) and total flavonoid content (TFC) as well as higher antioxidant activity than DW. Phytochemical screening demonstrated that all of the screening showed positive results in all extraction methods, except glycoside. There were no significant differences (*p* > 0.05) in TPC and TFC during MAE/ET, SE/ET, and UAE/ET. Antioxidant analysis shows that MAE and SE resulted in high (*p* < 0.05) DPPH and FRAP values for ET and DW, respectively. MAE/ET showed the highest inhibitory activity (IC_50_ = 16.67 µg/mL). HPLC and TLC analysis reveal the fingerprint of morin, which might function as an anticancer agent with other bioactives. Increasing the extract content increased the inhibitory activity of SW480 cells via MTT assay. In conclusion, MAE/ET is the most efficient among the extraction techniques in terms of anti-cytotoxicity effects.

## 1. Introduction 

Cancer is a serious life-threatening disease with a high death rate, and it is currently one of the leading causes of high mortality rates throughout the world [1]. There were more than 18 million cancer patients globally in 2020 with 9.3 million men and 8.8 million women. Death due to cancer reached 9.6 million in the year 2020 [2]. According to the most recent information from the International Agency for Cancer Research (IARC), 36 types of cancer have been documented in 185 countries around the world. Nearly half of the cancer patients live in Asia, the region with the largest incidence of cancer deaths. Colorectal cancer (CRC) is the third most often spotted cancer, behind lung and breast cancer, and ranked four in terms of the occurrence of death [3,4,5], accounting for more than 10% of overall cancer diagnosed and about 8% of overall death due to cancer [6]. Breast and lung cancers were the most frequent cancers worldwide in 2020, accounting for 12.5% and 12.2% of all new cases, respectively. CRC was the third most prevalent cancer in 2020, which accounted for 1.9 million cases and 10.7% of all new cases [2]. In recent times, the prevalence of CRC has been rapidly increasing. By 2035, there will be 1.36 million and 1.08 million cases of CRC in men and women, respectively, throughout the world [7]. In Thailand, there were 0.1906 million new cases of cancer in 2020, which counted men and women at 0.0934 and 0.0972 million cases, respectively. This contributed 11.1% CRC for both sexes and ages. However, 11.4% of CRC for males and 10.7% of CRC for females were diagnosed in 2020. By 2040, 0.29 million additional instances of cancer are predicted by the Global Cancer Observatory [8]. This knowledge makes it vital to research any potential treatments or medications for treating patients rather than using general treatments that were burdensome, complicated, and had adverse side effects.

According to epidemiological data, food and nutrition are crucial in the management and prevention of CRC [9]. Research suggests that dietary factors are attributed to 90% of CRC mortality [10], and consuming >400 g of fruits and vegetables per day is reported to cut the risk of CRC by 40% [11]. The bulk of plant bioactives are said to bind to dietary fiber, and fruits and vegetables are excellent sources of both bioactive chemicals and dietary fiber (DF). These bioactives are released into the colon as a result of colonic fermentation of DF by local probiotic bacteria. Plant-derived dietary bioactive compounds can be extremely helpful in the fight against cancer by preventing carcinogens from activating on target tissue and thereby reducing cancer formation. These phytochemicals are also thought to play a major role in secondary cancer prevention by lowering cell proliferation or increasing differentiation and death in tumor-initiating cells [12]. Several studies have been conducted to evaluate the possible anticancer effects of phytochemicals derived from a wide variety of fruit and vegetable extracts [13,14]. Some of the protective components present in fruits and vegetables include selenium, vitamins, and dietary polyphenols, such as flavonoids, phytoalexins, phenolic acids, indoles, carotenoids, and others [12,15]. The ability of these bioactive substances to exert their anticancer properties was discovered through in-depth research [16]. This has resulted in the findings of alternative cancer treatment approaches such as nutrition therapy, which fights cancer cells through a healthy diet without the adverse effects that patients frequently experience from conventional medicine. Hence, finding these bioactive chemicals, assessing their broad spectrum of pharmacological activity, and pinpointing their specific mechanism of action may aid in cancer therapy [17]. Numerous epidemiological studies have shown that dietary behaviors such as low fiber consumption, high-fat diets, and poor calcium and micronutrient intake are associated with colon cancer [18,19,20]. As a result, there is a growing tendency to concentrate on natural sources, such as plants and fruits, while looking for new anticancer drugs. The prevention and treatment of CRC may benefit from bioactive chemicals found in fruits and vegetables, including their waste products [21,22,23]. Peels and seeds of fruits and vegetables contain PCs that have the potential to be used as chemo-preventive and chemotherapeutic agents in CRC therapy [24]. 

PC substances having medicinal properties may be found in guava plant components including leaves, fruits, seeds, peels, pulp, bark, and oil. Guava leaf (GL) extract has been researched for potential chemotherapeutic use. Studies have demonstrated the antibacterial [25,26,27], anti-inflammatory [28], antimalarial [29], anticancer [30,31], and antiallergic [32] action of guava. GL, in particular, has the ability to inhibit different human carcinoma cell lines. GL contains quercetin and morin, two phenolic compounds with powerful antioxidants. Both are aglycones that have been used as dietary supplements and may be helpful in fighting a range of diseases. The prevention of cardiovascular disease; anticancer, anti-tumor, anti-ulcer, antiallergy, anti-viral, and anti-inflammatory activity; anti-diabetic and gastroprotective effects; and antihypertensive, immunomodulatory, and anti-infective effects are only a few of the benefits [33]. Shahid et al. [34] used HPLC and FTIR analysis to examine the chemical and antioxidant properties of 50% and 70% hydroethanolic Gola guava fruit (GGF) and guava leaf (GGL) extracts. Their study suggests GGL shows higher phenolic compounds and AA than GGF. The higher TPC and TFC were seen in the 50% hydroethanolic leaf extract, indicating that the solvent containing 50% hydroethanol was more effective in extracting bioactive chemicals. TPC was higher in GGL50% than GGL70%. The antioxidant activity (AA) of GGL50% and GGL70% was higher than that of GGF extracts. TPC demonstrated a strong positive association with DPPH, a somewhat positive correlation with H_2_O_2_, and a negative correlation with ABTS. HPLC analysis indicates that GGL50% shows higher phenolic compounds followed by GGF50%. Quercetin and kaempferol, as well as phenolic acids including Gallic, syringic, m-coumaric, *p*-coumaric, vanillic, ferulic, benzoic, caffeic, chlorogenic, cinnamic, sinapic, and ascorbic acids, were detected by HPLC analysis. Previous research has demonstrated the anticancer properties of quercetin and morin, which may lower the risk of cancer in general [35] and colorectal cancer in particular [36]. Arima and Danno [25] identified four antibacterial substances from guava leaves and used chemical and spectroscopic evidence to identify their structures. Morin-3-O-α-L-lyxopyranoside and morin-3-O-α-L-arabopyranoside, as well as two well-known flavonoids, guaijavarin and quercetin, were identified. Rattanachaikunsopon and Phumkhachorn [37] isolated four flavonoids from fresh and dried guava leaves, including morin-3-O-lyxoside, morin-3-O-arabinoside, quercetin, and quercetin-3-O-arabinoside. There is currently no one extraction technique that can be used to effectively extract PC from plant materials. When choosing an extraction method, there are several factors to consider, including extract quality, yield, costs, and consumer and environmental protection [38]. The use of traditional extraction techniques, such as solvent extraction, maceration, hydro or steam distillation, Soxhlet extraction, squeezing, or cold processing, is time consuming and requires a huge amount of solvent and energy [39]. As a result, there is a demand for innovative extraction techniques that need less solvent, less time, and produce higher extraction yields [40,41,42]. Nowadays, most of the food processing industries are searching for cutting-edge extraction techniques to reduce energy and costs, product or process safety, quality, and functionality. Non-thermal extraction methods such as ultrasound, supercritical fluid, and pulsed-electric-field-assisted extraction, as well as thermal ones such as microwave and pressurized liquid, are known to be very effective extraction techniques [43]. In comparison to conventional extraction procedures, these cutting-edge approaches have benefits of quicker extraction times, less energy use, lower costs, and less use of organic solvents [44,45,46]. A few of the solvents are used to extract phytochemicals from plant materials, including water [47], ethanol, hydroethanol [48] methanol [26], and hydro-methanol [49]. Nonetheless, there has been little research to find the best solvent for AA of GL. A few studies used green extraction techniques to extract bioactive chemicals from GL, including SE [50], MAE [51], and UAE [52]. Considering the above discussion, this research aimed to compare those green extraction techniques for the extraction of bioactive chemicals in guava leaf as well as to determine the anticancer and antioxidant effects of the extracts. In this research, phytochemical screening, TPC, and TFC of DW and ET extracts of GLs using SE, MAE, and UAE were assessed. The best extraction technique and solvent with high antioxidant and anti-cytotoxicity were also evaluated.

## 2. Materials and Methods

### 2.1. Chemicals

Trolox ((±)-6-Hydroxy-2,5,7,8-tetramethylchromane-2-carboxylic acid), DPPH (2,2-diphenyl-1-picryhydrazyl), TPTZ (2,4,6-Tri(2-pyridyl)-s-triazine), Iron(II) sulfate heptahydrate (FeSO_4_), Folin-Ciocalteu reagent, gallic acid (≥99% purity), sodium carbonate, methanol, ethanol, and tricholoacetic acid were bought from Merck (Darmstadt, Germany). Ferric chloride (FeCl_3_) was provided by Ajax Finechem (Seven Hills, Australia). Dulbecco’s modified Eagle’s medium (DMEM), MTT (3-(4, 5-dimethylthiazol-2-yl)-2, 5-diphenyl tetrazolium bromide), and L-glutamine Sigma-Aldrich Chemicals (St Louis, MO, USA) was bought from Sigma-Aldrich Chemicals (St Louis, MO, USA). Fetal bovine serum (FBS) was bought from Gibco-BRL (Auckland, New Zealand). Water (LC-MS grade) was provided by RCI Labscan (Bangkok, Thailand). 

### 2.2. Collection of Raw Materials 

Fresh pink GLs were collected from Kaset Natee Farm (Mae Chan, Chiang Rai, Thailand). Young-age green leaves were targeted; 1–3 leaves on top of each branch were collected for this study. Collected fresh leaves were washed with running tap water, followed by air-drying under light exposure until all water droplets present were totally evaporated. The core of the leaf was removed manually, and the leaves were dried at 40 °C for 12 h by a cabinet try dryer (BP80, KN Thai TwoOp, Bangkok, Thailand) followed by cryogenic grinding to powder using liquid nitrogen and a blender [53]. The powder was sieved through a 6.73 mm (3 mesh) sized sieve and stored in the refrigerator (4 ± 1 °C) using an HDP zipper (Ziploc^®^) until further use. 

### 2.3. Preparation of Sample Extract 

#### 2.3.1. Solvent Extraction (SE)

A total of 50 g of guava powder was mixed with 500 mL of DW and 50 mL of 60% ET. The solution was heated to 100 °C for 20 min before being shaken at 240 rpm for 6 h at room temperature (RT, 28 ± 2 °C) using an orbital shaker (KS130, Schwerte, Germany) [50]. The extract was filtered using a vacuum pump and Whatman No. 1 solvent-resistant filter paper after being centrifuged (MPW-352R, MPW MED. Instruments, Warszawa, Poland) at 8000 rpm for 10 min. A rotary evaporator (RV 3 V, Schwerte, Germany) at 50 °C and at 100 rpm was used to concentrate the acquired aqueous organic extract until 50 mL of the extract was obtained and the organic solvent was entirely evaporated. The extract was lyophilized in a freeze dryer (Labconco, FreeZone8L, MO, USA) after being frozen in dry ice until the samples were dry and stored at −40 °C for further analysis. 

#### 2.3.2. Microwave-Assisted Extraction (MAE) 

A total of 50 g of guava powder was mixed with 500 mL of DW and 50 mL of 60% ET. The suspension was subjected to magnetic stirring (WH220, Wiggens, Straubenhardt, Germany) for 45 min at 950 rpm at RM (28 ± 2 °C) followed by microwave heating at 800 W for 140 s. Initial heating for 40 s was followed by two consequent heating cycles of maximum 10 s each. A 40 s intermittent cooling time is maintained between any two heating cycles [51]. The extract was filtered by Buckner funnel using Whatman No. 4 solvent-resistant filter paper after being centrifuged (MPW-352R, MPW MED. INSTRUMENTS, Warszawa, Poland) at 8000 rpm for 10 min. A rotary evaporator (RV 3 V, Schwerte, Germany) at 50 °C and at 100 rpm was used to concentrate the acquired aqueous organic extract until 50 mL of the extract was obtained and the organic solvent was entirely evaporated. The extract was frozen in dry ice and lyophilized in a freeze drier (Labconco, FreeZone8L, MO, USA) until the samples were dry. The dried extract was kept in storage at −40 °C until further use. 

#### 2.3.3. Ultrasound-Assisted Extraction (UAE)

A total of 50 g of guava powder was mixed with 500 mL of DW and 50 mL of 60% ET. The suspension was subjected to magnetic stirring (WH220, Wiggens, Straubenhardt, Germany) for 45 min at 950 rpm at room temperature (28 ± 2 °C) and sonicated (CPX2800H-E, BRANSON ULTRASONICS CORPORATION, CT, USA) (ultrasound frequency 40 KHz at 404 W) at 62 °C for 20 min [52]. The extract was filtered by Buckner funnel using Whatman No. 4 solvent-resistant filter paper after being centrifuged (MPW-352R, MPW MED. Instruments, Warszawa, Poland) at 8000 rpm for 10 min. A rotary evaporator (RV 3 V, Schwerte, Germany) at 50 °C and at 100 rpm was used to concentrate the acquired aqueous organic extract until 50 mL of the extract was obtained and the organic solvent was entirely evaporated. The extract was frozen in dry ice and a lyophilized freeze drier (Labconco, FreeZone8L, MO, USA) until the samples were dry. The dried extract was kept in storage at −40 °C until further use. 

### 2.4. Phytochemical Screening 

A different test was performed using the standard techniques outlined below. 

#### 2.4.1. Tannins and Phenol 

In test tubes, 1 g of each sample was added individually to 20 mL of DW. The mixture was heated for 10 min in a water bath; the liquid was filtered into Erlenmeyer flasks while still hot using Whatman filter paper No. 1. Once the filtrate had cooled, 1 mL of it was diluted to a volume of 5 mL with DW, and 2–3 drops of 10% FeCl_3_ were then added to the mixture. The development of a bluish-black or brownish-green precipitate ensured the presence of tannins and phenols [54].

#### 2.4.2. Alkaloids

A total of 5 mL of aqueous extract was mixed with 2 mL of 2N HCl. Each mixture was constantly stirred for 10 min while being heated in a water bath. It was then cooled, followed by filtration. The filtrate was analyzed for alkaloids using a few drops of Dragendorff’s reagents [55]. The development of a reddish-brown precipitate indicated the presence of alkaloids.

#### 2.4.3. Saponins

A total of 10 ml of DW was mixed with 1 g of each sample. The solution was boiled in a water bath for at least 10 min and filtered through an Erlenmeyer flask while it was still hot. Foam and emulsion tests were determined after cooling. A test tube containing 2.5 mL of the filtrate was filled, diluted to a volume of 10 mL with DW, and shaken violently for 2 min in the foam test. The formation of foam makes sure that saponin is present in the filtrate. In the foam, 2 drops of olive oil were added, and the mixture was violently agitated for a few minutes. The formation of a rather stable emulsion indicates the existence of saponins [56].

#### 2.4.4. Terpenoids 

A total of 2 ml of chloroform was added to 5 mL of aqueous extract. To create a layer, 2 mL of concentrated H_2_SO_4_ was carefully added and gently shaken. The reddish-brown color in the inter-phase confirmed the presence of terpenoids [57].

#### 2.4.5. Glycosides 

A total of (2) ml of the aqueous extract was mixed with 1 mL of glacial acetic acid, 2–3 drops of FeCl_3_, and 2–3 drops of concentrated H_2_SO_4_. A green/blue precipitate was found to be present, which indicated the presence of glycosides [55].

#### 2.4.6. Amino Acids 

In 2 mL of aqueous extract, 5–6 drops of the ninhydrin reagent were added. The solution was heated for 5 min in a water bath. The presence of amino acids was shown by the purple coloration of the solution [55].

#### 2.4.7. Proteins 

In 2 mL of aqueous extract, 5–6 drops of 5% NaOH and 5–7 drops of 1% Cu(SO_4_)_2_ were added and mixed properly. The presence of proteins was indicated by the violet color [55].

### 2.5. Determination of Phenolic Compounds 

#### 2.5.1. Total Phenolic Compounds (TPC)

TPC was measured using the Folin–Ciocalteu technique described by Malik and Ahmad [58] with slight modifications. The sample was diluted (100-fold) by DW, and 1 mL was transferred into a test tube. A total of 5.0 mL of 10% *v*/*v* Folin–Ciocalteu’s reagent was added to the tube, followed by 4 mL of sodium carbonate solution (7.5% *w*/*v*). The suspension was rested for 1 h in a dark room at 28 ± 2 °C. A UV-visible spectrophotometer (Lambda 35 PerkinElmer, Bangkok, Thailand) was used to detect the absorbance at 765 nm. The standard curve of gallic acid, which ranges from 20 to 80 µg/mL, was used to calculate the content of polyphenols in the samples. The gallic acid equivalents (mg GAE/g) per gram of dry extract were used to measure the TPC.

#### 2.5.2. Total Flavonoid Content (TFC)

TFC was measured using AlCl_3_ methods [59]. For sample preparation, 0.1 g of the extract was transferred to a 100 mL flask, and the volume was made up of 100 mL of DW. A total of 2 mL of the solution and 2 mL of aqueous AlCl_3_·6H_2_O (0.1 mol/L) were mixed properly. The suspension was allowed to stand for 40 min in a dark room at 28 ± 2 °C. A UV-visible spectrophotometer (Lambda 35 PerkinElmer, Bangkok, Thailand) was used to detect the absorbance at 417 nm. Total flavonoid contents are calculated as milligram quercetin equivalents per gram of dried extract (mg QEs/g). 

### 2.6. Antioxidant Activity

#### 2.6.1. DPPH (2,2-Diphenyl-1-Picryl-Hydrazyl-Hydrate) Radical Scavenging Activity 

DPPH radical functions as a free radical or oxidizing radical that is decreased by antioxidants, as well as a reaction indicator. The DPPH activity was measured utilizing the stable radical 2,2-diphenyl-1-picryl-hydrazyl-hydrate by Brand-Williams’ technique [60] with slight modifications. A total of 50 µL of the extract that had been 100-fold diluted was mixed with 2000 µL of 60 mM DPPH radical in methanol. The mixture was vortexed for 20 s and rested for 1 h in a dark room at 25 °C for the reaction to occur. A UV-visible spectrophotometer (Lambda 35 PerkinElmer, Bangkok, Thailand) was used to detect the absorbance at 417 nm using methanol as a control. For the standard, 20–80 µg/mL of Trolox was prepared. The concentration of trolox (µg/mL) and the percentage of inhibition were used to plot the calibration curve. The amount of TEAC (Troxol equivalent antioxidant capacity) per gram of dried extract (mg TEAC/g) was used to represent the DPPH radical scavenging activity. 

#### 2.6.2. Determination of Ferric Reducing Antioxidant Power Activity (FRAP Assay)

Reducing activity was measured using the ferric reducing antioxidant power (FRAP) assay, which was modified from Benzie’s method [61]. The FRAP reagent was made by mixing 300 mM acetate buffer (pH 3.6), 10 mM TPTZ in 40 mM HCl, and 20 mM FeCl_3_ in a 10:1:1 (*v*/*v*/*v*) ratio. Standard ferrous sulfate (Fe (II)) was prepared (62.5–1000 µM). A total of 400 µL of standard and 100-fold diluted extract were mixed in 2600 µL of FRAP reagent. The mixer was incubated in a water bath for half an hour at 37 °C. A UV-visible spectrophotometer (Lambda 35 PerkinElmer, Bangkok, Thailand) was used to detect absorbance at 595 nm. The ferric reducing antioxidant power activity was measured in millimoles of Fe (II) equivalent per gram of dried extract (mmol Fe(II)/g). 

### 2.7. Cell Culture Treatment and Cell Viability by MTT Assay

American Type Culture Collection (ATCC, Manassas, VA, USA) provided the human colon cancer cells (SW480). The cancer cells were grown in Dulbecco’s modified Eagle’s medium (DMEM) supplemented with 10% FBS, 2 mN L-glutamine, and 100 IU penicillin/streptomycin. SW480 cells were grown on tissue culture plates that were 100 mm in diameter and incubated at 37 °C with 5% CO_2_ and 95% air atmosphere, and media were changed every 2–3 days. After reaching >80% confluence, the cells were trypsinized, collected, and transplanted onto a brand-new tissue culture dish. 

The anticancer activity was measured by the percentage of cell viability using the 3-[4, 5-dimethylthiazole-2-yl]-2, 2, 5-diphenyltetrazolium bromide (MTT) test. The viability of SW480 cells was assessed by the MTT assay of Mosmann [62]. The SW480 colon cancer cell line was seeded in a 96-well plate at a density of 4 × 10^4^ cells per well, and it was incubated at 37 °C with 5% CO_2_ and 95% air atmosphere. Different concentrations (12.5, 25, 50, 100, and 200 g/mL) of extracts were applied to the cells for 24 h. Then, each well received 20 µL of MTT (5 mg/mL). After 2 h of incubation, the supernatant was discarded. The cells were given two PBS washes before being exposed to 0.5 mg/mL of resazurin for 4 h. After 4 h, the cell viability was determined at 570 nm by a micro-plate reader (Multiskan FC, Thermo Fisher Scientific, Waltham, MA, USA). The following equation was used to calculate the percent cell growth inhibition.

(1) % of cell growth inhibition ((ODofuntreatedcells)−(ODoftreatedcells))/   ODofuntreatedcells×100

### 2.8. HPLC-MS Analysis for Identifying Morin Compound 

Chromatographic studies were carried out (DAD) using an HPLC Agilent 1260 series (Agilent Technologies, Santa Clara, CA, USA) outfitted with a binary pump, an online degasser, an autosampler, a thermostatically controlled column compartment, and a UV-Vis Diode Array Detector. The column temperature was maintained at 25 °C. Bioactive compounds of guava leaf extract were separated at 28 ± 2 °C using a modified method of López-Cobo et al. [63]. Phenolic compounds were separated using a Poroshell 120 EC-C18 (4.6 mm × 100 mm, 2.7 m particle size, Agilent Technologies, Santa Clara, CA, USA). The gradient elution was carried out using acetonitrile as the solvent system B and water with 1% acetic acid as the solvent system A. The following procedures were followed: 0 min, 0.8% B; 2.5 min, 0.8% B; 5.5 min, 6.8% B; 11 min, 14.4% B; 17 min, 24% B; 22 min, 40% B; 26 min, 100% B; 30 min, 100% B; 32 min, 0.8% B; and 34 min, 0.8% B. The sample volume was maintained at 5 µL with a flow rate of 0.8 mL/min. 

MS analysis was performed using Agilent 6540 Ultrahigh-Definition Accurate-Mass Q-TOF-MS connected to an HPLC, furnished with an Agilent Dual Jet Stream electrospray ionization (Dual AJS ESI) lined in negative ionization mode. The following conditions were used for MS analysis: N_2_ flow rate = 120 L/min, nebulizer pressure = 50 psi, gas drying temperature = 370 °C, capillary voltage = 3500 V, fragmentor voltage = 3500 V, and scan range, m/z 50–1500. In automated MS/MS studies, the following collision energy values were used: m/z 100, 30 eV; m/z 500, 35 eV; m/z 1000, 40 eV; and m/z 1500, 45 eV. Data elaboration and integration were accomplished using the Mass Hunter Workstation program (Agilent Technologies, Santa Clara, CA, USA) [64]. 

### 2.9. TLC Analysis 

Thin-layer chromatography (TLC) was used to evaluate the extracts using several eluents and detection solutions [65]. TLC analysis was carried out using 5 mg of extract diluted in 1 mL of ethyl acetate. Silica gels (5 × 20 cm) were used for sample separation. Using hexane: ethyl acetate (80:20) as the eluent, essential oils, flavonoids, and antioxidants were separated from the extract. Essential oils are identified using an anisaldehyde solution. Flavonoids are isolated using NP/PEG or boric acid and oxalic acid in ethanol. 

## 3. Results and Discussion 

### 3.1. Phytochemical Screening 

This study was conducted to identify some of the major secondary metabolites found in the leaves of *Psidium guajava* Linn, including tannins, phenols, saponins, flavonoids, steroids, terpenoids, and alkaloids. The phytochemical screening indicated that tannins, phenols, flavonoids, alkaloids, saponins, and terpenoids were identified in the extract. However, glycosides were not identified in the GL extract as shown in Table 1. Amino acids and proteins were also detected in the guava leaf extract. Research revealed that the protein content of GL is 9.7% (dry basis) [66]. Thomas et al. [67] found that GL contained 8.4 mg of amino acids and 16.8 mg of protein per 100 g measured by ninhydrin and Lowry’s methods, respectively. Since GL is a high source of proteins, carbohydrates, and dietary fibers, it can be used as a novel and sustainable food source [68]. GL is a very good source of several macro- and micronutrients, as well as bioactive compounds, which are beneficial for human health. GL has a moisture content of 82.47%, 103 mg of ascorbic acid, 3.64% ash, 0.62% fat, 18.53% protein, and 1717 mg gallic acid equivalents (GAE)/g total phenolic compounds [69]. Kim et al. [70] found several health-promoting carbohydrates in GL such as fucose, rhamnose, arabinose, galactose, glucose, mannose, and xylose. Fucose is crucial for host–microbe interactions, selectin-mediated leukocyte-endothelial adhesion, and blood transfusion responses [71]. GLs are abundant in minerals such as calcium, potassium, sulfur, sodium, iron, boron, magnesium, manganese, and vitamins C and B. GLs are an excellent choice for human nutrition and animal feed to reduce micronutrient deficits due to their enhanced Mg, Na, S, Mn, and B concentrations [72]. According to Thomas et al. [67], 100 g of GL contains 1660 mg Ca, 360 mg P, 1602 mg K, 13.50 mg Fe, and 440 mg Mg per 100 g of GL (dry weight). It also contains 103 mg and 14.80 mg per 100 g DW of vitamins C and B, respectively. 

Several researchers conducted a qualitative phytochemical analysis of GL extracts extracted by macerating GL powder in ethanol. Biswas et al. [57] detected phenols, tannins, terpenoids, flavonoids, and glycosides but no saponins in *Psidium guajava* L. extracts. Terpenoids, quinones, lipids, and phenol are present in the GL powder extracts, but alkaloids, flavonoids, sterols, and anthocyanin are not detected [73]. Geoffrey et al. [74] identified alkaloids, flavonoids, phenols, and tannins in the *Psidium guajava* leaf extracts but failed to identify saponins, steroids, terpenoids, or cardiac glycosides from Kericho and Baringo Counties, Kenya. The presence of alkaloids, glycosides, saponins, and tannins was detected but no flavonoids or steroids were identified in the *Psidium guajava* leaf extract prepared by ethanol purification at 30 °C [75]. *Psidium guajava* leaf extract contains phlobatannins, saponin, flavonoids, steroids, terpenoids, polyphenols, and glycosides but not triterpenoids, alkaloids, or anthraquinone, suggested by Thenmozhi and Rajan [76]. The results demonstrated that all of the screenings gave a positive result in all extraction methods, except glycoside, which gave a negative result for all three extraction methods. Phytochemical screening analysis suggested that there is a difference in the level of the result between extraction methods and solvent used for the extraction (Table 1). PC screening of *P. guajava* Linn leaf extracts revealed varying degrees of presence of secondary metabolites. The solvent extraction process shows a strong presence of total flavonoids, total phenolic, alkaloids, and terpenoids in both DW and ET. However, only saponins, tannins, and phenols are less present in DW and ET for the solvent extraction method. The UAE process shows a strong presence of total flavonoids, alkaloids, saponins, and terpenoids in ET, and total phenolic, tannins, and phenols had a strong presence in the case of DW. However, total phenolic, tannins, phenols, and terpenoids had a strong presence in the ET process for the MAE method. Most of the phytochemicals were less present, shown in the MAE method for DW, except tannins and phenols. The presence of secondary metabolites in aqueous extracts was almost similar, although the intensity of color was less, which might be attributable to the fact that some compounds may not be properly soluble in aqueous solvents [77]. ET extracts of GL had a better presence of secondary metabolites than water extracts except for tannins and phenols (Table 1).

Several studies on plant parts show that flavonoids are probably responsible for pharmacological and biochemical activities, including antioxidant, antiallergic, anti-inflammatory, hepatoprotective, anti-carcinogenic, anti-viral, and anti-thrombotic properties [78]. Tannins are used in treatments that are anti-hemorrhoidal, hemostatic, and anti-diarrheal. Saponins function as anti-inflammatory and antioxidant substances that help to decrease cholesterol [78]. Terpenoids have a major role in wound healing, skin strength, wound antioxidant concentration, and the capacity to heal inflamed tissues by boosting blood flow [79]. PCs have several biological activities including the inhibition of angiogenesis and cell proliferation, cardiovascular protection, anti-apoptosis, anti-inflammation, anti-aging, anti-atherosclerosis, anti-carcinogen, and the improvement of endothelial function. Steroids show antibacterial properties and are very important compounds due to their interactions with other substances such as sex hormones [80]. 

### 3.2. Effect of Extraction Techniques on Percent Yield of the Extract

The findings indicated that UAE/ET (10.37%) had the greatest yield, followed by SE/ET (9.45%), SE/DW (7.95%), MAE/ET (7.11%), MAE/DW (6.85%), and UAE/ET (6%) (Table 2). The ET gave the highest extraction yield for all three extraction processes. Research revealed that increasing the polarity of the solvent increased the extraction yield [81]. Water is a fairly good solvent, although this is due to the fact that water is primarily a polar solvent and will only dissolve polar molecules. Since ethanol is polar but also includes a significant non-polar component, it may frequently dissolve both polar and non-polar molecules, not simply polar ones. As a result, the higher percentage yield shown by the ET may be attributable to the ability to dissolve both polar and non-polar molecules, which may allow it to extract a vast range of compounds. According to the foregoing findings, the efficient extraction of bioactive compounds from GL is highly dependent on the solvent type during extraction. Different types of solvents with various polarities extract specific phytochemicals from plants [82]. In this study, ET (hydroethanol) yielded the highest amount of crude extract with the highest presence of phytochemicals. As a result, this investigation supports the concept that differences in solvents used will affect the presence of bioactive chemicals in an extract [83]. It also denotes that the selection of a solvent is affected by several aspects, such as the class of phytochemicals, diversity, and polarity of the compounds to be extracted [84]. 

The findings indicated that UAE/ET had the greatest yield (*p* < 0.05), followed by SE/ET (9.45%) and MAE/ET (7.11%). Chuyen et al. [39] discovered that the UAE had much higher (*p* < 0.05) maximal carotenoid and antioxidant capacity yields than the MAE. The antioxidant activity of the UAE extract was likewise much higher (*p* < 0.05) than that of the traditional extraction using the same solvent-to-material ratio. The results revealed that both MAE and UAE may be utilized to greatly shorten the extraction time of Gac peel when compared to traditional extraction while still achieving high extraction efficiencies. By rupturing plant cell walls, ultrasounds can speed up the transmission of heat and mass, which improves the release of desired chemicals from a range of natural sources [85]. The maximum extraction yield was 3.13%, demonstrating the effectiveness of the ultrasonic technique for extracting antioxidants from black mulberry fruits in a ratio of water: materials 40:25, using ultrasonic power 190 W for 735 min at 69 °C. Wang et al. [86] reported that at 70 °C, 230 W of power, and a water to material ratio of 13:1 mL/g, the highest extraction (5.16%) was observed for the ultrasound-assisted antioxidant activity of pears. This technique is quite effective for the food industry. Corbin et al. [87] extracted phenolic compounds from pine seeds using ultrasound technology. The technique is very effective for reducing the trapping of phenolic compounds with 0.2 N NaOH, extraction for 60 min, at 25 °C, and an ultrasonic frequency of 30 KHz, which increases the extraction of bioactive compounds by 30% as opposed to conventional maceration.

### 3.3. Effect of Solvent on the Extraction of Phytochemicals

Extraction is a critical step in producing extracts rich in phenolic compounds. The type of solvent, extraction temperature, and time are all factors that impact phytochemical extraction. Phytochemicals are frequently extracted using ethanol, methanol, acetone, and water. Nevertheless, no suitable solvent is used for the isolation of whole components. However, no appropriate solvent is employed for the separation or isolation of whole phenolic compounds. Water, ethanol, and hydroethanol are the most often utilized extraction solvents in food systems due to their abundance, affordability, and compatibility with health [88]. Ethanol is utilized for phytochemical extraction, according to the laws regarding the use of food-grade solvents [89]. Water, hydroethanol, and low concentrations of ethanol can enter cells very quickly and easily, while high concentrations of ethanol can promote protein denaturation, inhibiting polyphenol dissolution and then influencing the extraction rate [90]. Water is a strong polar solvent, whereas ethanol is a low-polar solvent with which any amount may be mixed [91]. The addition of water to ethanol raises the polarity of the complex solvent gradually. Since the molecules of phenolic compounds are polar in nature, according to the “like dissolves like” principle, the yield of TPC rose as the water concentration increased [92].

Table 2 demonstrated that ET produced the highest values in all test results. TPC and TFC concentrations were higher in ET than DW. This finding is consistent with the findings of Qian and Nihorimbere [48], who found that the water extract of guava leaves contained fewer phenolic compounds than the 50% hydroethanolic extract. This is due to the co-extraction of both polar and less polar molecules [93]. Another investigation found that the 40% hydroethanolic extract had the greatest phenolic component level [94]. In comparison to water alone, pure ethanol, or methanol, ethanol–water mixtures generally demonstrate better phenolic component extraction efficiency, especially those containing 40 to 80% ethanol [95]. According to Seo et al. [96], the hydrophenolic extracts had greater phenolic component contents than water extracts, with the 50% hydroethanolic extract having the greatest phenolic compound contents. According to Nyirenda et al. [97], polar molecules such as flavonoids and other polyphenols are more soluble in aqueous solvents than in organic solvents. Diaz-de-Cerio et al. [64] observed that the phenolic content of pure ethanol extracts was lower than that of extracts obtained using hydro-alcoholic mixtures because of the lower solubility of polar chemicals in pure organic solvents. Hydroethanol (40% water and 60% ethanol, *v/v*) enhances phytochemical extraction since the target chemicals are more soluble in solvent systems [96]. 

### 3.4. Effect of Extraction Techniques on Phenolic Content 

The TPC of the extracted solution from three extraction methods is evaluated by the Folin–Ciocalteu method and reported as milligram Gallic acid equivalents per gram of dried extract (mg GAE/g). Table 2 displays the TPC extracted by DW and ET using different extraction methods. The calibration curve using gallic acid exhibited maximum absorbance at 765 nm (y = 0.00886 + 0.127, R^2^ = 0.996). ET shows significantly (*p* < 0.05) higher TPC than DW for all extraction techniques, i.e., MAE, UAE, and SE. Among the three extraction methods, SE/ET demonstrates the highest (66.0 mg GAE/g) amount of TPC followed by MAE/ET (65.29 mg GAE/g) and UAE/ET (63.23 mg GAE/g). However, all three methods were not significantly (*p* > 0.05) different from each other in terms of TPC extracted by ET. The UAE/ET technique produced the lowest TPC values. The possible causes for this include less solvent penetration into the solute matrix, which would lead to a lower yield and lower phenols in the extract [98]. SE/DW (58.89 mg GAE/g) had a higher (*p* < 0.05) TPC value, followed by UAE/DW (54.47 mg GAE/g) and MAE/DW (46.70 mg GAE/g). The possible causes for this include higher solvent penetration into the solute matrix, which would lead to a higher yield and higher phenols in the extract [98]. The results of TFC in the extracted solution from three extraction methods are shown in Table 2. The equation for the quercetin standard calibration curve was y = 0.00192 + 0.0881, R^2^ = 0.999. Similar to TPC, ET shows significantly (*p* < 0.05) higher TFC than DW for all extractions techniques, i.e., MAE, UAE, and SE. Among the extracted solutions from three extraction methods, SE/ET contained the highest (129.01 mg QE/g) amount of TFC followed by UAE/ET (125.13 QE mg/g) and MAE/ET (123.69 mg QE/g). However, all three methods were not significantly (*p* > 0.05) different from each other in terms of TFC extracted by ET. SE/DW (96.18 mg QE/g) had the highest TFC followed by UAE/DW (86.54 mg QE/g) and MAE/DW (75.35 mg QE/g). Though, UAE/DW and MAE/DW did not significantly differ (*p* > 0.05) from each other based on TFC. This might be due to the coupled effect of SE, which allows for better solvent penetration into the solid matrix. For TPC and TFC, ET extraction shows better results than only DW. The best reason behind this is described in Section 3.2.

Flavonoids and other plant phenolic compounds, which have strong antioxidant capabilities, can trap free radicals and reactive oxygen species [99]. One of the most important aspects of obtaining high-quality natural antioxidants is the extraction procedure. This research shows that GL is a good source of antioxidant phyto-compounds such as morin, and its glycosides, quercetin and guaijaverin. In this study, several extraction methods such as MAE, UAE, and SE were used to assess their impact on phytochemical and antioxidant profiles. Given its high efficiency for the extraction of plant bioactives, the SE methodology has proven to be the best extraction method, followed by MAE and UAE in the case of DW extraction. However, MAE/ET, UAE/ET, and SE/ET show the same impact on the extraction of TFC and TPC. The UAE utilized green extraction methods as a basis because of a phenomenon called “cavitation”, which happens when strong shear pressures and free radicals combine to damage the cell wall. When used in conjunction with electromagnetic microwaves for consistent heating, these methods provide greater efficiency and yields [100]. The extremely high frequency of ultrasonication was expected to disrupt the structure of the plant cell wall, resulting in enhanced contact between the solvent and the plant material. As a result, the breakdown of active components was accelerated in this approach [101]. In the instance of ET, the findings showed that MAE had a similar extraction capability to TPC and TFC to SE and UAE (*p* > 0.05). However, SE shows significantly higher extraction of TPC and TFC than UAE and MAE for DW. Although the extraction of TPC and TFC by MAE is similar (*p* > 0.05) to that of UAE and SE in the case of ET, the treatment time for MAE was shorter. The higher extraction of TPC and TFC with a shorter extraction time might be attributed to ionic conduction and water dipole rotation, which are the primary mechanisms of microwave heating. Plant materials are effectively provided by molecular interactions with the electromagnetic field as pressure builds up inside the cells of a sample, and energy is swiftly transmitted to the extraction solvent and raw plant materials [94]. As pressure builds up inside the cells, plant materials are efficiently delivered through molecular interactions with the electromagnetic field, and energy is quickly transferred to the extraction solvent and raw plant materials [102]. The results of this study are expected to be helpful for both micro- and macro-scale commercial extraction of natural antioxidants from guava leaves.

### 3.5. Effect of Extraction Techniques on Antioxidant Activity (AA) 

The DPPH radical scavenging and FRAP antioxidant power assays were used to assess the AA of the extracted solution from GL using three extraction procedures (MAE, UAE, and SE) (Table 2). It is demonstrated that MAE/ET showed the highest DPPH free radical scavenging capacity (43.44 mg TE/g), followed by SE/ET (43.06 mg TE/g), and UAE/ET (41.50 mg TE/g). Between the three techniques, there was, however, no statistically significant difference (*p* > 0.05). AA research using DPPH and FRAP revealed that ET extraction had much stronger AA than only DW (Table 2). In comparison to UAE/DW (35.79 mg TE/g) and MAE (33.081.13 mg TE/g), SE/DW had a significantly (*p* < 0.05) better DPPH scavenging activity (39.85 mg TE/g). Since AA is directly connected to reducing capacity, the FRAP assay is a reliable approach for assessing antioxidant activity in extracts by donation of electrons (Fe^+3^ to Fe^+2^). The FRAP radical scavenge results are shown in Table 2. The FRAP followed the same pattern as the ability to scavenge radicals using the DPPH free radical assay. The result suggested that MAE/ET shows better scavenging ability (45.14 mmol Fe(II)/g), followed by SE/ET (45.10 mmol Fe(II)/g) and UAE/ET (44.37 mmol Fe(II)/g). Between the three techniques, there was, however, no statistically significant difference (*p* > 0.05). Similar to DPPH, the FRAP scavenging activity of SE/DW (41.50 mmol Fe(II)/g) shows higher antioxidant activity (*p* < 0.05) than UAE/DW (38.45 mmol Fe(II)/g) and MAE/DW (34.79 mmol Fe(II)/g). The AA was shown to be much higher in the ET for all measurements. The higher phenolic content indicates a positive correlation between phenolic component concentration and AA. Increasing the extract concentration increased the antioxidant activity (Table 2). 

Since ethanol extract contains more antioxidants than water, it has the ability to dissolve more polar molecules than a water solvent. The findings revealed that the AA as well as total phenolic and total flavonoid contents of sample extracts were in the same order (ET > DW). This outcome is consistent with research by Jagadish et al. [103], which demonstrated a substantial link between bioactive substances and AA. The results of this study are consistent with previous studies that investigated the relationship between phenolic compounds and AA. An earlier study discovered that AA was influenced by the profile of the phenolic component [104]. Kim et al. [105] found a positive correlation between AA and phenolic compound concentration. Additionally, Seo et al. [96] found that hydroethanolic extracts had superior AA than water extracts and that 50% hydroethanolic extract had the maximum antioxidant activity. Additionally, Qian and Nihorimbere [48] discovered that water extract had lower AA than 50% hydroethanolic extract. It is suggested that the quantity of phenolic compounds was strongly connected with the DPPH- and ABTS+ scavenging activities [106]. Huang et al. [107] observed that the activity began to decline with increasing concentration after a critical point and ascribed this to interference from other compounds. Our findings strongly suggest that the AA of GL, including their capacity to reduce free radicals and scavenge DPPH and FRAP, is highly reliant on the presence of phenolic compounds. The AA based on DPPH and FRAP was higher in extracts using MAE/ET compared to SE/ET and UAE/ET. However, no significant (*p* > 0.05) variations in AA were found. The quick breakdown of plant cells by electromagnetic waves after exposure to microwave heating may be the cause of the higher TPC in MAE. When compared to standard extraction procedures, MAE offers several distinct advantages, including quicker extraction times, greater extraction yields, and reduced solvent usage [108].

### 3.6. The Correlation Analysis

**Figure 1:**
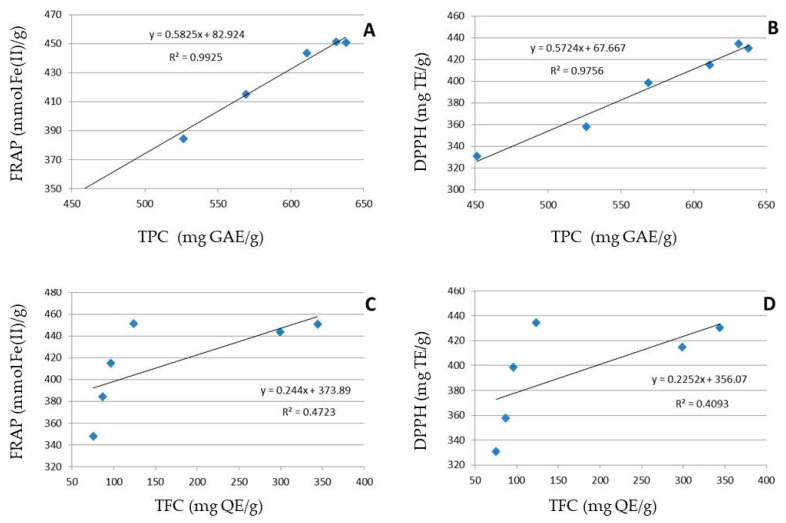
The correlation between TPC and FRAP (**A**), TPC and DPPH (**B**), TFC and FRAP (**C**), and TFC and DPPH (**D**). DPPH = 2,2-diphenyl-1-picrylhydrazylhydrate; FRAP = Ferric reducing antioxidant power; TPC = Total phenolic content; TFC = Total flavonoid content equivalent; TE = Torolox equivalent. The data are means ± SD of 3 replicates.

Some studies have found a linear relationship between total phenolic and flavonoid concentration and antioxidant capacity [109]. Figure 1 illustrates the correlation between total phenolic and flavonoid content with AA. Numerous studies have found a linear relationship between TPC, TFC, and AA [109]. Figure 1 illustrates the correlation between TPC, TFC, and AA. There were strong correlations between AA and TPC (DPPH, R^2^ = 0.9756; FRAP, R^2^ = 0.9925) and a moderate correlation with TFC (DPPH, R^2^ = 0.4093; FRAP, R^2^ = 0.4723) at a 95% confidence level. By analyzing the correlation coefficients (R-values), it is feasible to conclude that the phenolic and flavonoid groups have a significant impact on the AA of the selected plant extracts. 

TPC shows a linear relationship with the AA of all extracts. Meanwhile, TFC was shown to have a non-linear relationship with the AA of all extracts, with an increase in TPC and/or TFC in guava leaves, increasing antioxidant activity measured by DPPH and FRAP techniques. A previous study suggested that condensed tannin in GLs may have contributed to AA [110]. This finding is consistent because condensed tannin is a phenolic substance. Research revealed that GL includes essential oils that are abundant in cineol, triterpenes, tannins, eugenol, kaempferol, and other substances such as flavonoids, malic acid, gallic acid, chlorophyll, and mineral salts [111]. However, according to other studies, the main components of guava leaves include rutin, naringenin, gallic acid, catechin, epicatechin, kaempferol, isofavonoids, and favonoids including quercetin and guaijaverin [17,112]. For all three extraction procedures (SE, UAE, and MAE), the TPC in ET was higher than the TPC in DW, with higher DPPH and FRAP values. This indicates that many phenolic compounds in ET can transfer hydrogen more efficiently than DW. 

### 3.7. Extract Analysis 

#### 3.7.1. HPLC-MS

**Figure 2:**
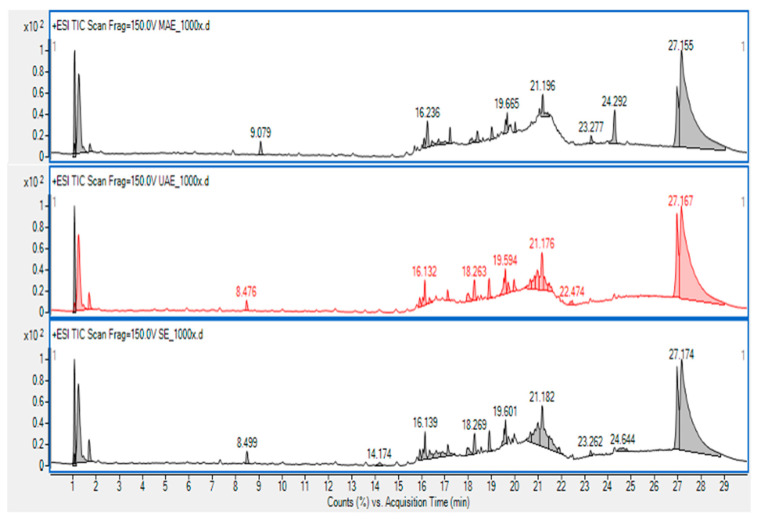
HPLC-MS chromatogram of MAE (**Top**), UAE (**Middle**), and SE (**Bottom**). The data are means ± SD of 3 replicates.

The HPLC profile analysis of *Psidium guajava* Linn. leaf extract showed the comparative profile of three different extracted methods, including MAE (top), UAE (middle), and SE (bottom) (Figure 2). Morin is one of the flavonoids primarily identified in the extract of GLs. The pro-oxidative impact of morin in colon cancer cells (SW480 cells) causes a disruption in mitochondrial activity, which activates the intrinsic and extrinsic apoptotic pathways [113]. Morin also causes a significant reduction in glucose transporter-1 expression, which lowers cellular glucose absorption. Additionally, mitochondrial activity is impaired by morin, which makes cells more susceptible to death via the intrinsic apoptosis pathway [113]. According to Figure 2, the peak where UAE (middle) and SE (bottom) were absent may be morin, which is most present in MAE (top) related to the anti-cytotoxicity effect on MTT assay. This can demonstrate that MAE had the highest anti-cytotoxicity activity. Rattanachaikunsopon and Phumkhachorn [28] isolated four flavonoids from both fresh and dried *Psidium guajava* leaves, including morin-3-O-lyxoside, morin-3-O-arabinoside, quercetin, and quercetin-3-O-arabinoside. However, quercetin and morin-3-O-arabinoside were the most and least common substances, respectively. 

Arima and Danno [18] also isolated morin and its glycosides, morin-3-O-α-L-lyxopyranoside and morin-3-O-α-L-arabopyranoside, from GL in addition to quercetin and its glycoside, guaijaverin (quercetin-3-O arabinoside) [18]. Díaz-de-Cerio et al. [64] found 72 bioactive compounds, morin being one of them, from commercial GL using ET (70: 30, *v/v*) and analyzed by HPLC-DAD-QTOF-MS. Similarly, Díaz-de-Cerio et al. [114] discovered 48 phenolic compounds, morin being one of them. Morin is one of the phytochemicals exhibiting a wide range of biological and/or pharmacological activities with very low cytotoxicity and is well known for its AA [115]. Morin belongs to the flavonol groups and one of the major sources of morin is different parts of guava [116]. It acts as a protector against the oxidation of cell components by scavenging free radicals. The existence of a double bond between C2-C3 atoms and a hydroxyl group (-OH), which activates the double bond at the C-3 position, is the main cause of the antioxidant potential of morin [117].

#### 3.7.2. Thin-Layer Chromatography (TLC)

**Figure 3:**
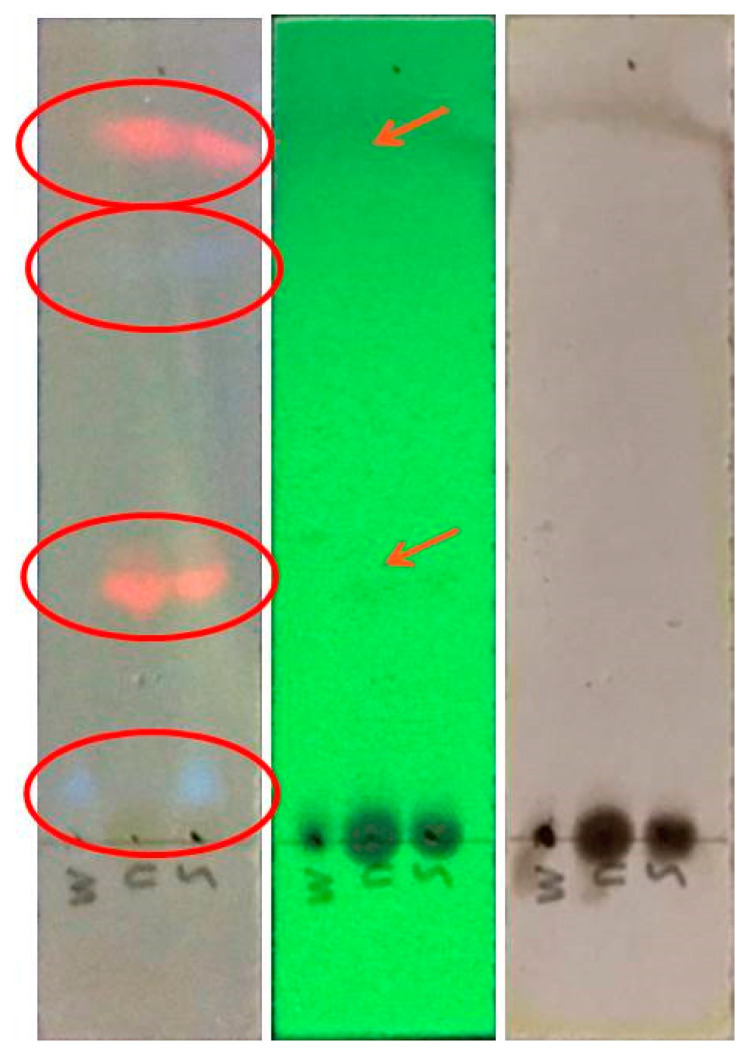
TLC chromatogram under Visible light (370 nm), UV light (254 nm), and dyed with Para-anisaldehyde, from left to right. M = Microwave-assisted extraction; U = Ultrasound-assisted extraction; S = Solvent extraction. Red circle and arrow indicates the presence or absence of peaks.

TLC was used to detect the presence of several types of chemicals in the extracts and estimate their relative abundance (Figure 3). The chemicals that are active under UV light were identified by exposing TLC plates to UV light shortly after elution. Morin (one of phytochemicals present in GL, as discussed in Section 3.7.1) was detected in all of the extraction techniques used in this investigation. 

**Figure 4:**
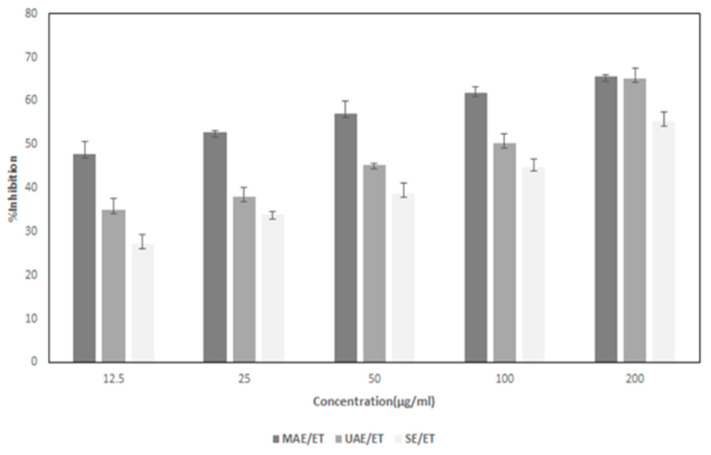
% inhibition of SW480 colon cancer cells via MTT assay of *Psidium guajava* Linn. leaf extract. SE = Solvent extraction, UAE = Ultrasound-assisted extraction; MAE = Microwave-assisted extraction. The data are means ± SD of 3 replicates.

The highest amount of antioxidants was found in MAE/ET extracts, which also included significant quantities of morin. The higher flavonoid content of the MAE extracts may also have contributed to the higher concentration of antioxidant molecules. After the TLC plate was developed under visible light and UV light and covered with para-anisaldehyde, the peak was clearly observed on the TLC plate. Figure 4 indicates that the peaks of MAE (M) are absent; maybe the compounds in this extraction method are higher or near the mobile phase. On the other hand, the peak compounds in UAE (U) and SE (S) were present; maybe the polar molecules in both methods were nearby polar molecules with the mobile phase. 

### 3.8. Cytotoxicity Activity of the Extracts 

The MTT experiment demonstrated that GL extract (12.5–200 µg/mL), which is supposed to be morin (morin, one of the phytochemicals in GL, was detected by HPLC-MS and TLC), lowered the survival rate of SW480 cells in a time- and dose-dependent manner, which revealed that GL extract (might be morin) exhibits anti-cytotoxicity ancer properties (Figure 4). ANOVA analysis suggests that the effects of dosage and time on cell viability were shown to be statistically significant (*p* < 0.05). Table 3 shows the inhibitory effects of GL extract (might be morin) on the SW480 cancer cell (only this cell was used because of the limitation of our laboratory at this time). Moreover, the MTT experiment demonstrated that the inhibitory effect of GL extract (might be morin) extracted by different extraction methods shows significantly different (*p* < 0.05) inhibitory effects. 

Higher AA was shown by a lower IC_50_ value, which also suggested a greater capacity to donate hydrogen. When compared to DW, all ET extraction of GL had a higher capacity to scavenge DPPH and FRAP radicals. This study indicates that GL extract (might be morin) extracted by MAE/ET showed stronger AA than UAE/ET and SE/ET. The best AA was shown by MAE (16.67 µg/mL) followed by UAE (98.68 µg/mL) and then SE (144.59 µg/mL) (Table 3). Increasing the GL extract concentration increased the % inhibition (Figure 4).

According to the current study, morin (according to HPLC-MS and TLC analysis) from GL extract exhibits dose-dependent activity. A few research studies also found that GL extracts had dose-dependent efficiency. The MTT assay was used to determine the cytotoxicity against liver carcinoma cells, and the results showed that the extract had a dose-dependent inhibitory activity against HepG2 cell growth, with cell viability of 81.85%, 70.65%, 53.19%, and 31.09% after exposure to 5, 20, 50, and 100 g/mL, respectively [118]. Peng et al. [118] investigated the antiangiogenic and antimetastatic efficacy of another aqueous extract of *P. guajava* budding leaves on DU145 cells. A dose-dependent mode was observed by the GL extract during the inhibition of DU145 cell survival, which shows an IC50 of 0.57 mg/mL. The above results support the hypothesis that aqueous extracts of *P. guajava* budding leaves have a potent anti-prostate cancer action. The findings of this study also indicated that MAE extract demonstrated considerably higher % inhibition (*p* < 0.05) than UAE and SE, except at 200 µg/mL. At the highest concentration, MAE and UAE extract exhibit similar % inhibition. MAE extracts indicated better % inhibition and IC50, which might be due to the purity of the extract [119]. The use of the microwave is a flourishing technology since it allows easy, safe access to temperature and reproducibility, as well as decreases response times, increases yields, and improves purity, when compared to conventional heating techniques [119]. However, because of the numerous factors that might influence MAE, the extraction process parameters must be optimized to extract the highest amount of phenolic compounds and AA [120]. Indeed, microwave power is one of the important factors influencing polyphenol release from various matrices by rupturing cell walls, and it also has the ability to influence equilibrium and mass transfer conditions during extraction. By increasing microwave power, the extraction of polyphenols can be accelerated [120].

This study shows that GL extract (morin is one of the bioactives present in GL extract) demonstrated a chemo-preventive effect on SW480 cells in a time- and dosage-dependent manner. Apoptosis is a critical mechanism that results in cell death in order to regulate cell growth. Compounds that induce apoptosis have been thought to have therapeutic potential in anticancer treatment [121,122]. Cells treated by morin inhibit the dimethylhydrazine (DMH)-induced NF-κB pathway and inflammatory cytokines COX-2, IL-6, TNF-, and PGE-2. It is possible that Morin has a pro-apoptotic effect since it drastically reversed the DMH-induced Bax and Bcl-2 ratio [122]. Morin successfully decreased the growth of aberrant crypt foci (ACF) and depressed the activity of mucosal and fecal bio-transforming enzymes in DMH-treated rats [123]. An in vitro study revealed that morin inhibits the proliferation of human colorectal cells as well as the formation of colorectal cancer in vivo [124]. Morin remarkably inhibits colon carcinogenesis, lowers the risk of colon cancer, and reduces the colon neoplasms size [125]. According to Lori et al.’s research [126], long-term morin therapy significantly decreased the expression of low-molecular-weight protein tyrosine phosphatase (LMW-PTP) and significantly decreased the number of colon precancerous lesions. Morin treatment has the potential to increase chemotherapy sensitivity and stop the development of cancer [17]. Finally, it may be inferred that there is a strong possibility that morphin can trickle colorectal cancer via a different pathway. The probable mechanism of cancer cell proliferation involves caspase 3-mediated Poly(ADP-ribose) Polymerase (PARP) breakage, as assessed by the use of a downstream caspase 3 inhibitor [126]. Morin treatment of SW480 cells resulted in caspase 3-mediated PARP breakage. Cell death was decreased when cells were treated with a downstream caspase 3 inhibitor prior to morin exposure, indicating that morin plays a role in cell death [126]. The levels of cleaved caspase 3 and cleaved PARP were similarly significantly reduced after pretreating SW480 cells with a caspase 3 specific inhibitor, demonstrating that caspase 3 activation plays a major role in morin-induced cell death in SW480 colon cancer cells [126].

## 4. Conclusions

Phytochemical screening indicated that guava leaf was positive for a class of secondary metabolites; however, glycoside tested negative. This study found that UAE/ET yielded the maximum extraction yield (10.37%). Additionally, hydroethanolic extracts contain more phenolic components than water extracts. Hydroethanolic extracts have more antioxidant activity than water extracts. However, TPC and TFC did not significantly differ (*p* > 0.05) in the case of SE/ET, UAE/ET, and MAE/ET. The results of antioxidant activity indicated that the highest DPPH (43.44 mg TE/g) and FRAP (45.14 mmol Fe(II)/g) radical scavenging activity was observed in MAE/ET method. The phenolic components in guava leaves mostly contribute to their antioxidant activity through DPPH and FRAP techniques. HPLC-MS and TLC analysis suggests that morin, an aglycone phenolic compound, has been extracted successfully from the leaves of *Psidium guajava* Linn. The MTT assay analysis shows that MAE (IC_50_ = 16.67 µg/mL) is the best method to extract morin from guava leaves. A dose-dependent manner was observed for the % inhibition of colon cancer cells (SW480) via the MTT assay. In conclusion, microwave-assisted hydroethanol extraction might be suggested for the extraction of bioactive compounds that have anti-cytotoxicity effects. However, further research should focus on the effect of GL extracts on normal human cells or using any other assays for the inhibition of cell proliferation or migration. The anticancer activity of guava leaf extracts found in in vitro studies should be replicated by in vivo studies for confirmation and underlying molecular pathways. Furthermore, in vivo animal research with higher dosages and clinical trials with human participants are required to determine its safety in humans.

## Figures and Tables

**Table 1:** Qualitative phytochemical screening of extracted solution of *Psidium guajava* Linn. leaves.

Class of Compounds	MAE		UAE		SE	
Solvent	Distilled water	60% ethanol	Distilled water	60% ethanol	Distilled water	60% ethanol
Total flavonoid	+	++	++	+++	+++	++++
Total phenolic	+	+++	+++	++	++++	++++
Tannins and Phenols	++++	+++	+++	++	+++	+
Alkaloids	+	++	++	++++	+++	++++
Saponins	+	++	+	+++	+	+++
Terpenoids	+	++++	++	++++	+++	++++
Glycosides	-	-	-	-	-	-
Protein	+	++	+	++	+	++
Amino acids	+	++	+	++	+	++

The screening results are based on 3 replicates. SE = Solvent extraction, UAE = Ultrasound-assisted extraction; MAE = Microwave-assisted extraction. + = positive result; - = negative result; and the number of + and - signal showed the level of result.

**Table 2:** Yield, total phenolic content, total flavonoid content, and antioxidant activities of guava leaf extract.

Extraction Method	Solvent	% Yield	TPC (mg GAE/g)	TFC (mg QE/g)	DPPH (mg TE/g)	FRAP (mmol Fe(II)/g)
MAE	Distilled water	6.85 ± 0.06 ^e^	46.70 ± 3.75 ^d^	75.35 ± 3.12 ^e^	33.08 ± 1.13 ^d^	34.79 ± 1.56 ^d^
	60% ethanol	7.11 ± 0.08 ^d^	65.29 ± 1.62 ^a^	123.69 ± 11.77 ^ab^	43.44 ± 0.61 ^a^	45.14 ± 1.24 ^a^
UAE	Distilled water	6.00 ± 0.06 ^f^	54.47 ± 2.21 ^c^	86.54 ± 7.81 ^d^	35.79 ± 0.12 ^c^	38.45 ± 0.87 ^c^
	60% ethanol	10.37 ± 0.06 ^a^	63.23 ± 2.91 ^ab^	125.13 ± 4.95 ^a^	41.50 ± 1.21 ^ab^	44.37 ± 1.06 ^a^
SE	Distilled water	7.95 ± 0.04 ^c^	58.89 ± 3.07 ^b^	96.18 ± 1.17 ^c^	39.85 ± 0.58 ^b^	41.50 ± 0.92 ^b^
	60% ethanol	9.45 ± 0.13 ^b^	66.00 ± 1.21 ^a^	129.01 ± 9.52 ^a^	43.06 ± 1.04 ^a^	45.10 ± 0.32 ^a^

The data are means ± SD of 3 replicates. Mean values with different letters in the same column are significantly different (*p* < 0.05). DPPH = 2,2-diphenyl-1-picrylhydrazylhydrate; FRAP = Ferric reducing antioxidant power; TPC = Total phenolic content; TFC = Total flavonoid content; GAE = Gallic acid equivalent; QE = Quercetin equivalent; TE = Torolox equivalent; SE = Solvent extraction, UAE = Ultrasound-assisted extraction; MAE = Microwave-assisted extraction.

**Table 3:** Effect of extraction techniques on the growth inhibition and IC_50_ of colon cancer cells via MTT assay.

Extraction Method	Concentration (µg/mL)	Growth Inhibition (%)	IC_50_ (µg/mL)
MAE	12.5	47.83 ± 2.94	16.67 ± 3.65 ^c^
	25	52.73 ± 0.47	
	50	57.12 ± 2.74	
	100	61.59 ± 1.24	
	200	65.51 ± 0.35	
UAE	12.5	35.05 ± 2.65	98.68 ± 0.8 ^b^
	25	37.91 ± 2.13	
	50	45.31 ± 0.33	
	100	50.20 ± 2.11	
	200	65.10 ± 2.41	
SE	12.5	27.11 ± 2.33	144.59 ± 22.30 ^a^
	25	33.89 ± 0.72	
	50	38.78 ± 2.26	
	100	44.83 ± 1.89	
	200	55.19 ± 2.26	

The data are means ± SD of 3 replicates. Mean values with different letters in the same column are significantly different (*p* < 0.05). SE = Solvent extraction, UAE = Ultrasound-assisted extraction; MAE = Microwave-assisted extraction.

## Data Availability

Data are contained within the article.
